# Comparative Analysis of Surgical Fixation Techniques for Pediatric Odontoid Fractures: A Systematic Review

**DOI:** 10.7759/cureus.88866

**Published:** 2025-07-27

**Authors:** Razan Zantout, Imad Ashkar, Rawan Masarwa, Kawthar El Khatib, Reem Aldanaf, Sam Najjar, Carelle Karam, Rebecca Bou Kanj, Alain El Marji, Neel Badhe, Chinedu Egu, Elie Najjar

**Affiliations:** 1 Gilbert and Rose-Marie Chagoury School of Medicine, Lebanese American University, Jbeil, LBN; 2 Centre for Spinal Studies and Surgery (CSSS) Queen's Medical Centre, Nottingham University Hospitals, Nottingham, GBR; 3 School of Medicine, University of Cambridge, Cambridge, GBR

**Keywords:** odontoid, odontoid fractures, pediatrics, surgical fixation, surgical outcomes

## Abstract

This review evaluates anterior, posterior, and combined fixation techniques for pediatric odontoid fractures, addressing the lack of comparative data on outcomes and complications.

A Preferred Reporting Items for Systematic Reviews and Meta-Analyses (PRISMA)-based review identified 1,497 studies, with eight meeting inclusion criteria after screening. Studies were assessed for quality using the Joanna Briggs Institute checklist, and data on demographics, fracture types, surgical techniques, fusion rates, and complications were analyzed.

Among 62 pediatric cases, posterior approaches were the most common (66.1%), followed by anterior approaches (27.4%) and combined approaches (6.5%). Fusion rates were highest with combined approaches (100%), followed by the posterior approach (92.7%) and the anterior approach (88.2%). However, the posterior approach had the highest complication rate (43.9%), including infections, persistent symptoms, and failed fusion. Combined approaches demonstrated superior outcomes with no complications, whereas anterior techniques, although effective in select cases, resulted in one mortality.

This review highlights the superior outcomes of combined approaches for complex pediatric odontoid fractures, achieving 100% fusion with no complications. Posterior techniques remain reliable, but advancements in technique and postoperative care are necessary to reduce complications further. High-quality, multicenter studies are crucial for establishing standardized management protocols and evaluating long-term outcomes.

## Introduction and background

Pediatric odontoid fractures are significant injuries due to their impact on cervical spine stability, neurological function, and the unique anatomical considerations in children. In a multicenter study of 165 pediatric spinal trauma cases, only two odontoid fractures were reported, making them significantly less frequent compared to other spinal injuries such as vertebral compaction fractures (234 cases, 78%) and burst fractures (25 cases, 8%) [1]. These fractures often occur through the synchondrosis cartilage between the odontoid process and the axis body, especially in children under eight years old. The developing spine, with its higher elasticity and incomplete ossification, presents challenges for both diagnosis and management, requiring careful surgical planning [2]. Clinicians may face difficulty distinguishing normal developmental ossification centers of the C2 vertebra from true fractures, making it essential to understand the age-dependent anatomy and common injury patterns of the C2 arch in children to avoid misinterpretation and ensure accurate diagnosis and appropriate management [3]. Pediatric cervical spine anatomy is fundamentally different from that of adults, with characteristics such as a cartilaginous odontoid synchondrosis, underdeveloped bony structures, and open growth plates, all of which make standard fixation techniques more technically demanding and potentially hazardous [4]. These anatomical distinctions increase the complexity of treating odontoid fractures in children and necessitate customized surgical strategies to avoid injury to growth centers, ensure stability, and promote safe healing [4]. Given these challenges, a comparative analysis of surgical techniques is essential to determine which methods best accommodate the unique biomechanical and developmental factors present in the pediatric cervical spine.

**Figure 1:**
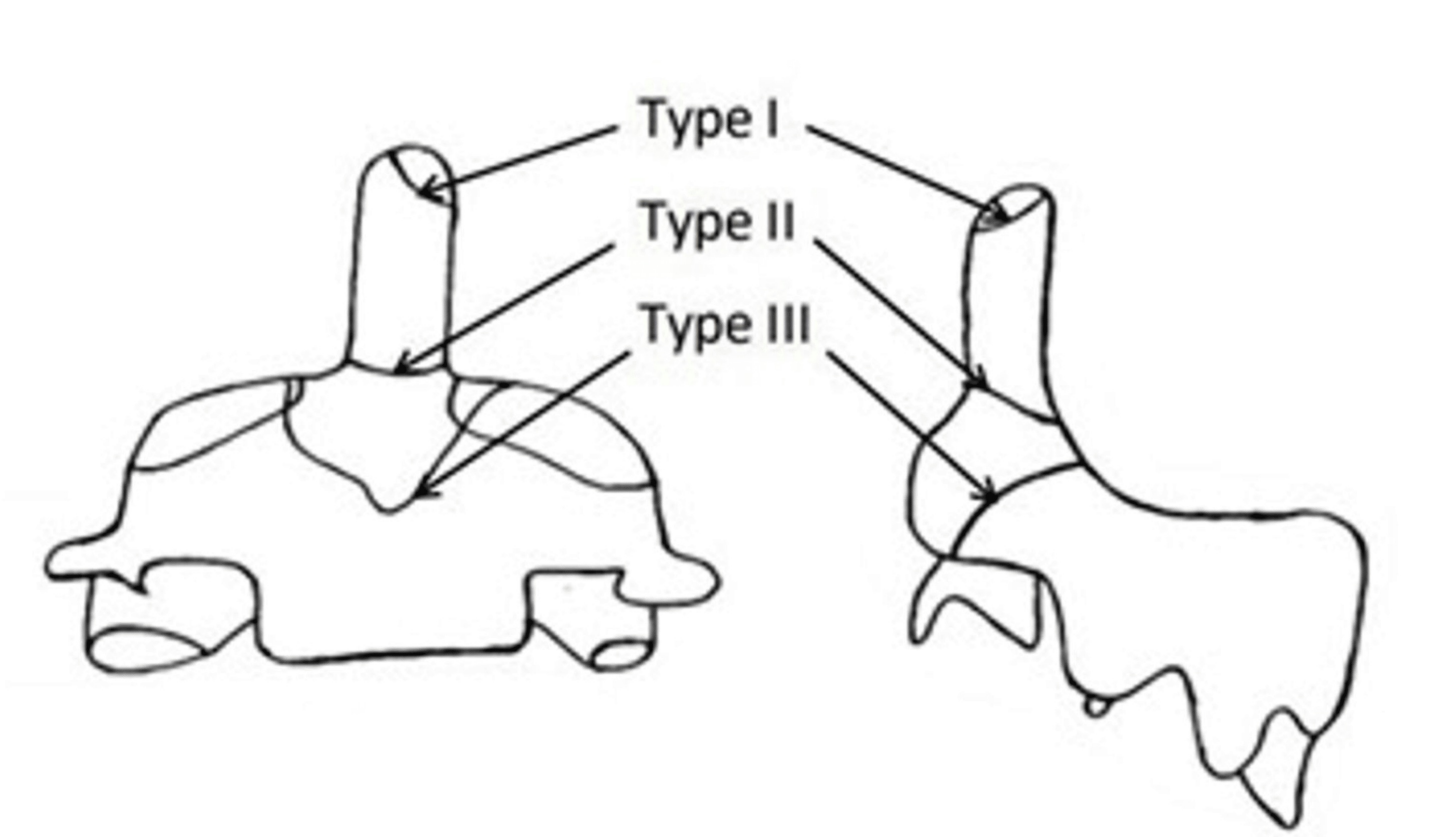
Anderson and D’Alonzo classification of odontoid fractures Image Credit: Authors

The mechanism of injury typically involves high-energy trauma, such as motor vehicle collisions, and patients may present with severe posterior cervical pain, tenderness, dysphagia due to retropharyngeal hematomas, or neurological symptoms like paresthesia and limb weakness [5,6]. The Anderson and D'Alonzo classification, which categorizes fractures into type I (tip), type II (base), and type III (extension into the vertebral body), remains the gold standard for guiding management (Figure 1) [7].

Management options for pediatric odontoid fractures include both conservative and surgical approaches. Conservative treatments, such as halo fixation or rigid cervical collars, are generally reserved for stable fractures or younger children where fusion is more likely. Surgical intervention becomes necessary in cases of instability, non-union risk, or neurological compromise [6,8].

Maak and Gauer emphasized that fracture type plays a critical role in determining the surgical approach [8]. For type I fractures, which are generally stable, conservative management is preferred, with surgery rarely indicated unless associated with ligamentous instability. Type II fractures, being the most common and unstable, often necessitate surgical fixation. Here, the anterior approach using odontoid screws is ideal when fracture anatomy allows for direct stabilization, preserving rotation and minimizing surgical morbidity. However, in cases where direct screw placement is not feasible due to anatomical constraints or the presence of comminution, posterior fixation becomes the approach of choice. For type III fractures that extend into the vertebral body, posterior fixation is generally favored, offering robust stability and promoting fusion through constructs such as C1-C2 wiring or transarticular screws [8]. However, posterior C1-C2 fixation in young children with odontoid fractures can hinder normal cervical spine development by stunting the growth of the C2 vertebral body, potentially leading to subaxial alignment deformities [9].

While individual case series describe outcomes for specific surgical techniques, a lack of comprehensive comparative data remains to evaluate their relative effectiveness in the pediatric population. Understanding how surgical approaches differ in fusion rates, complication profiles, and reoperation needs is essential for improving preoperative decision-making and optimizing patient outcomes. The choice of technique directly influences postoperative stability, preservation of range of motion, and potential neurologic recovery, making comparative analysis clinically relevant. Furthermore, pediatric surgical research faces unique challenges, including ethical limitations, anatomical variability during growth, and small patient populations that preclude the conduct of randomized trials or large cohort studies. Acknowledging these factors is crucial for interpreting the existing evidence and informing clinical decisions.

Despite advances in surgical instrumentation and techniques, few studies provide a comprehensive comparison of these approaches in the pediatric population. Therefore, this systematic review seeks to address this critical gap in the literature by synthesizing available evidence on fusion success, complication rates, and surgical indications across anterior, posterior, and combined approaches in pediatric odontoid fractures.

## Review

Methodology

Search Strategy

This systematic review was conducted following the Preferred Reporting Items for Systematic Reviews and Meta-Analyses (PRISMA) guidelines [10]. Comprehensive literature searches were conducted using PubMed and Web of Science for studies published up to August 2024, focusing on odontoid fractures in the pediatric population. The search spanned the period from inception to August 23, 2024. Grey literature and additional databases such as Embase, Scopus, and the Cochrane Library were not included in the search strategy. The keywords and search combinations used are the following: ((paediatric*) OR (Child) OR (Children) OR (“Pediatrics”[Mesh])) AND ((odontoid) OR (axis) OR (dens) OR (C2) OR (Odontoideum) OR (“Odontoid Process”[Mesh])) AND ((fracture) OR (“Fractures, Bone”[Mesh])).

Inclusion and Exclusion Criteria

Studies were included if they reported postoperative outcomes and surgical fixation techniques (anterior, posterior, or combined approaches) for children under 18 years with odontoid fractures. Eligible study designs included observational studies, case series, cohort studies, or randomized controlled trials. Exclusion criteria included non-English studies, expert opinion studies, case reports, conference abstracts, studies focused on conservative treatment, cases involving odontoid fractures with significant comorbidities (e.g., Down syndrome), and studies for which access could not be obtained. Before exclusion, efforts were made to obtain full-text articles through interlibrary services or by contacting the study authors.

Quality Assessment

Study quality was assessed using the appropriate JBI critical appraisal checklist for each study design. Each item was scored as "yes," "no," or "unclear" based on the information reported. "Unclear" was assigned when insufficient detail was provided to judge the item. Two authors conducted the quality assessments independently and in duplicate, with disagreements resolved through discussion or a third author. As shown in Table 1, the quality of included studies was appraised using the Joanna Briggs Institute (JBI) checklist for case series [11].

**Table 1:** Results of quality scoring based on the JBI appraisal checklist for the included studies JBI: Joanna Briggs Institute, Y: yes, N: no, U: unclear, Q: question

Number	Author	Q1/Q2	Q3/Q4	Q5/Q6	Q7/Q8	Q9/Q10	Total score
1	Odent et al., 1999 [12]	Y/Y	Y/Y	Y/Y	Y/Y	Y/U	90%
2	Meyer et al., 2001 [13]	Y/Y	Y/Y	Y/Y	Y/Y	Y/U	90%
3	Wang et al., 1999 [14]	Y/Y	Y/Y	Y/Y	Y/Y	Y/U	90%
4	Gao et al., 2023 [4]	Y/Y	Y/Y	Y/Y	Y/Y	Y/Y	100%
5	Varshney et., al, 2022 [15]	Y/Y	Y/Y	Y/Y	Y/Y	Y/U	90%
6	Lowry et al., 1997 [16]	Y/Y	Y/Y	Y/Y	Y/Y	Y/U	90%
7	Yang et al., 2018 [17]	Y/Y	Y/Y	Y/U	Y/Y	Y/U	80%
8	Sawarkar et al., 2021 [18]	Y/Y	Y/Y	Y/Y	Y/Y	Y/U	90%

Data Extraction

After the initial database search, duplicates were removed. All articles were independently screened by two authors, with any disagreements resolved through discussion or consultation with a senior author, followed by detailed, independent full-text assessments. Data extraction was conducted manually onto a predefined Excel spreadsheet (Microsoft Corp., Redmond, WA, USA).

The following data were extracted for each included study: first author and year of publication, number and types of odontoid fractures, mean age, sex distribution, surgical approach and techniques used, mean follow-up duration, fusion outcomes and complications, including persistence of symptoms, neurological decline, deep wound infections, failed fusion, kyphotic or scoliotic deformity, and mortality.

Statistical Analysis

The extracted data were analyzed using GraphPad Prism V10 software (GraphPad Software, Inc., San Diego, CA, USA), and missing data were handled by contacting the original author. Fisher's exact test was employed to evaluate correlations between categorical variables, such as fusion rates, persistence of symptoms, neurological decline, infections, failed fusion, kyphotic deformity, scoliosis, mortality, complications, and reoperations. For comparisons between independent groups with small expected cell counts, Fisher’s exact test was used instead of the chi-square test, as it provides a more accurate estimation under these conditions. Descriptive statistics were used to calculate means and percentages. No adjustment was considered for multiple comparisons. Due to anticipated clinical and methodological heterogeneity across studies, we did not plan or perform formal subgroup analyses, given the significant heterogeneity across studies and small sample sizes in the statistical analysis methods section.

Additionally, the limited number of eligible studies and small overall sample size precluded any meaningful subgroup comparisons. As such, all findings are presented as observational and exploratory. Meta-analysis was not conducted due to substantial clinical and methodological heterogeneity across studies, including variations in study design, populations, interventions, and outcome measures.

Results

Included Literature

**Figure 2:**
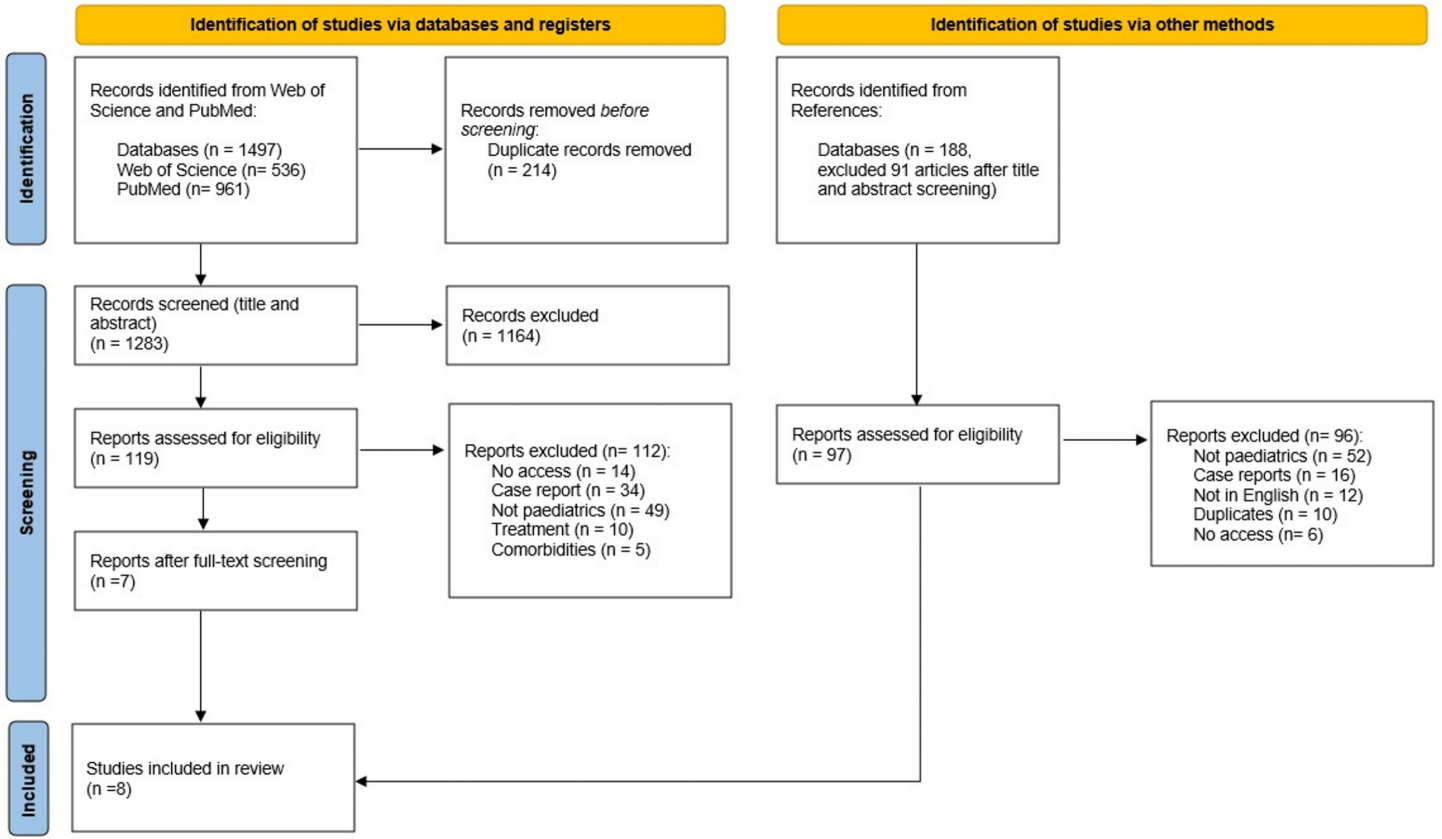
PRISMA flowchart PRISMA: Preferred Reporting Items for Systematic Reviews and Meta-Analyses

The initial search yielded 1,497 studies. After removing 214 duplicates, 1,283 titles were screened, leaving 119 studies for abstract and full-text evaluation. Of these, seven studies met the inclusion criteria. An additional 188 studies identified from references were screened, resulting in 97 full-text evaluations and one additional qualifying study. In total, eight studies were included in this review (Figure 2). Due to the significant heterogeneity among the included studies, a meta-analysis was not conducted. In addition, no saturation limit was applied in the selection of studies, and all eligible studies meeting the inclusion criteria were considered for analysis.

A total of 62 cases were reviewed across these studies (32.2% female, 58% male). Diagnoses included os odontoideum (27 cases), odontoid fractures (33 cases), and C1-C2 instability (2 cases). Surgical approaches included posterior (66.1%, n=41), anterior (27.4%, n=17), and combined (6.5%, n=4) techniques. The included studies encompassed a range of diagnoses, which were managed using different surgical approaches, such as the posterior, anterior, or combined, depending on the specific pathology and clinical context.

Surgical Approaches and Techniques

It is important to note that fusion was variably defined across the included studies. In some cases, assessment was made through the absence of motion on flexion-extension radiographs and the presence of calcifications, representing a clinical rather than a biological definition. Others defined fusion as radiographic evidence of bridging bone across the fracture line, often confirmed by CT or plain radiographs. Some studies combined imaging findings with clinical stability, while others assessed fusion solely through postoperative imaging. However, several studies did not explicitly define how fusion was defined, limiting the direct comparison of outcomes across the literature.

Posterior approach: This approach primarily utilized three techniques: (1) C1-C2 wiring (31.7%, n=13), which achieved fusion in 84.6% of cases, but complications were common, including deep wound infections (7.7%), delayed unions (15.4%), and neurological decline (7.7%); (2) transarticular screws (24.4%, n=10), which demonstrated a high fusion rate of 90%, but persistent symptoms were reported in 20% of cases; and (3) C2 pedicle or laminar screws (39%, n=16), which showed excellent fusion outcomes (100%) with no reported complications in this subset, highlighting the effectiveness of advanced instrumentation. Fusion rates ranged from 67% to 100% (mean: 85.4%). However, the posterior approach had the highest complication rate (43.9%, n=18). Common complications included persistent symptoms (19.5%), failed fusion (9.76%), deep wound infections (9.76%), and neurological decline (4.88%). Three patients (7.32%) required reoperation. Table 2 shows the summary.

**Table 2:** Study characteristics (posterior) M: male, F: female, N/A: not applicable

S/N	Study ID (first author, year)	n of odontoid fractures	Types of odontoid fractures	Mean age (range) years	n of M/F	Technique	Mean follow-up (months)	Complications	n of patients with complications
1	Meyer et al., 2001 [13]	6 (out of 8)	Post-traumatic os odontoideum (4), congenital os odontoideum (1), subacute type 2 odontoid fracture (1)	10 (3-16)	2/4	Halifax, Brooks, occipitocervical fixation (2), three-point fixation (2)	25.4 (12- 48)	5: neurological decline (2), persistence of symptoms (3)	3
2	Wang et al., 1999 [14]	6 (out of 9)	Os odontoideum posttraumatic (1), os odontoideum congenital (3), C1-C2 instability axis fracture (2)	9.4 (3-15)	5/1	C1-C2 screw/Sonntag	25 (3- 65)	5: persistence of symptoms (4), fusion extension to C2-3 (1)	4
3	Gao et al., 2024 [4]	4 (out of 7)	Displaced odontoid synchondrosis fracture	2.68 (1.75-4.34)	1/3	Midline incision and screw placement (C1 lateral mass screws and C2 pedicle screws)	34.7 (25-51)	0	0
4	Odent et al., 1999 [12]	3	Odontoid non-union, odontoid fracture	2.5 (0.8-5.5)	2/1	Posterior fusion with wiring of C1-C2 (3), Minerva (3)	51 (3-168)	5: delayed union (1), deep wound infection (1), recurrent displacement (1), fusion only at C2-3 (1), graft resorption (1)	3
5	Varshney et al., 2022 [15]	3	Unilateral atlantoaxial spondyloptosis with type-II odontoid fracture	11.25 (7-15)	3/0	Joint manipulation (3); joint remodelling (3)	36 (12-48)	2: persistence of symptoms (2)	2
6	Lowry et al., 1997 [16]	13	Type II odontoid fracture (2), os odontoideum (10), os odontoideum with vertebral artery dissection (1)	9.4 (3-16)	8/5	Brooks-type fusion (9), Gallie technique (1), Brooks w/ multistranded cable (3)	13 (4-32)	11: halo pin site infection (3), early halo removal (1), failed fusion (3), graft resorption (1), pin moved (1), persistence of symptoms (2), re-operation (3)	6
7	Yang et al., 2018 [17]	6	Os odontoideum	12.6 (5.2-17.8)	N/A	C2 translaminar screws (5 cases bilateral and one case on the left)	57.6 (8.4-127.2)	0	0

Anterior approach: This approach employed odontoid screw fixation, allowing direct stabilization of type II, IIA, and III fractures while preserving cervical rotation. Fusion was achieved in 88.2% (n=15) of cases. Complications were observed in 17.6%, including persistent symptoms, failed fusion, and one mortality (5.88%) due to neurovascular injury. One patient experienced transient difficulty with swallowing, which resolved spontaneously. Table 3 shows the summary.

**Table 3:** Study characteristics (anterior) M: male, F: female

S/N	Study ID (first author, year)	n of odontoid fractures	Types of odontoid fractures	Mean age (range) years	n of M/F	Technique	Mean follow-up (months)	Complications	n of patients with complications
1	Sawarkar et al., 2021 [18]	13	Posterior oblique or horizontal type II/IIA/III odontoid fractures/high type	15 (6-18)	11/2	K wire fixation and odontoid screw	36 (20-72)	2: death (1), non-union (1)	2
2	Meyer et al., 2001 [13]	1 (out of 8)	Acute type II odontoid fracture	10 (3-16)	0/1	Ventral odontoid screw fixation wiring C1-C2	25.4 (12-48)	0	0
3	Wang et al., 1999 [14]	3 (out of 9)	Type II odontoid fractures	9.4 (3-15)	3/0	Odontoid screw, K-wire	25 (3-65)	1: transient swallowing difficulty (1)	1

Combined approach: The combined approach involved anterior decompression, such as transoral odontectomy, followed by posterior fixation with C1-C2 screws or three-point fixation. Achieving a 100% fusion rate, this approach reported no complications, mortality, or reoperations. It was particularly effective for complex cases requiring multi-directional stability. However, given the limited sample size, these excellent outcomes should be interpreted with caution. However, given the limited sample size, these excellent outcomes should be interpreted with caution. Table 4 shows the summary.

**Table 4:** Study characteristics (combined) M: male, F: female

S/N	Study ID (first author, year)	n of odontoid fractures	Types of odontoid fractures	Mean age (range) years	n of M/F	Technique	Mean follow-up (months)	Complications	n of patients with complications
1	Gao et al., 2024 [4]	3 (out of 7)	Displaced odontoid synchondrosis fracture	2.68 (1.75-4.34)	0/3	Combined anterior release via a right-sided submandibular retropharyngeal approach and posterior fixation using C1-C2 screws	34.7 ± 8.5 (25-51)	0	0
2	Meyer et al., 2001 [13]	1 (out of 8)	Post-traumatic os odontoideum (1)	10 (3-16)	1/0	Transoral odontectomy followed by three-point fixation	25.4 (12-48)	0	0

Complications Summary

Across all surgical approaches, complications varied significantly (Table 5): persistence of symptoms occurred in 19.5% of posterior cases, 17.6% of anterior cases, and 0% of combined cases; failed fusion was observed in 9.76% of posterior cases, 5.88% of anterior cases, and 0% of combined cases; deep wound infection was reported in 9.76% of posterior cases, but 0% in both anterior and combined approaches; neurological decline occurred in 4.88% of posterior cases and 0% in the others; and mortality was 5.88% in the anterior approach, with 0% in posterior and combined approaches.

**Table 5:** Postoperative complications of all included studies

S/N	Study ID (first author, year)	Persistence of symptoms	Neurological decline	Deep wound infection	Failed fusion (non-union, displacement, metal work failure)	Kyphotic/scoliotic deformity	Re-operation	Death	Other
1	Sawarkar et al., 2021 [18]	0	0	0	1	0	0	1	Fibrous union but considered acceptable union (1)
2	Meyer et al., 2001 [13]	3	2	0	0	0	0	0	0
3	Wang et al., 1999 [14]	4	0	0	0	0	0	0	Transient swallowing difficulty (1), fusion extension to C2-3 (1)
4	Gao et al., 2024 [4]	0	0	0	0	0	0	0	0
5	Odent et al., 1999 [12]	0	0	1	2	0	0	0	Graft resorption (1), fusion of C2-3 (1), delayed fusion (1)
6	Varshney et al., 2022 [15]	2	0	0	0	0	0	0	0
7	Lowry et al., 1997 [16]	2	0	3	3	0	3	0	Early halo removal (1), graft resorption (1), pin moved (1)
8	Yang et al., 2018 [17]	0	0	0	0	0	0	0	0

Overall Outcomes

The combined approach yielded the most favorable results with a 100% fusion rate and no complications; however, these findings should be interpreted with caution due to the limited sample size. Posterior fixation, while achieving high fusion rates (85.4%), presented the highest complication burden. Anterior fixation demonstrated intermediate outcomes with a strong fusion rate (88.2%) but included a mortality case. These findings underscore the importance of tailoring surgical strategies to the type of fracture and patient-specific factors to optimize outcomes (Table 6).

**Table 6:** Summary statistics OR: odds ratio, CI: confidence interval, N/A: not applicable

	Anterior		Posterior		Combined		p-value (OR; 95% CI)
	No.	%	No.	%	No.	%	
Number of fractures	17/62	27.4	41/62	66.12	4/62	6.45	N/A
Fusion	15/17	88.2	35/41	85.4	4/4	100	p>0.9999
Persistence of symptoms	3/17	17.6	8/41	19.5	0/4	0	p>0.9999
Neurological decline	0/17	0	2/41	4.88	0/4	0	p>0.9999
Deep wound infection	0/17	0	4/41	9.76	0/4	0	p=0.4752
Failed fusion	1/17	5.88	4/41	9.76	0/4	0	p>0.9999
Kyphotic/scoliotic deformity	0/17	0	0/41	0	0/4	0	p>0.9999
Mortality	1/17	5.88	0/41	0	0/4	0	p=0.3387
Number of patients with complications	3/17	17.6	18/41	43.9	0/4	0	p=0.0597
Reoperation	0/17	0	3/41	7.32	0/4	0	p=0.6314

Discussion

There was significant heterogeneity across the included studies in terms of patient characteristics, with reported mean ages ranging from 0.8 to 17.8 years for the posterior approach, 3 to 16 years for the anterior approach, and 1.75 to 16 years for the combined approach. Additionally, there was considerable variation in fracture types, including post-traumatic os odontoideum, congenital os odontoideum, C1-C2 instability, axis fractures, and other cervical spine pathologies.

The treatment of odontoid fractures, os odontoideum, and C1-C2 instability in pediatric patients remains a complex challenge due to unique anatomical considerations, limited surgical experience, and variability in outcomes. This review highlights the outcomes and complications of posterior, anterior, and combined surgical approaches, offering insight into optimizing management strategies for this demographic.

The posterior approach was the most commonly used (66.1%, n=41). Fusion rates were high (67% to 100%), consistent with benchmarks in the field, indicating the reliability of posterior fixation in achieving stable outcomes. However, the complication rate of 43.9% exceeds the average reported in similar studies, highlighting the need for targeted strategies to mitigate risks such as infections and hardware-related issues. This higher complication burden, despite its frequent use, may be partly attributed to the anatomical complexity of the craniocervical junction, where posterior instrumentation places critical neurovascular structures such as the vertebral artery, brainstem, and spinal cord at risk. Additionally, posterior approaches often require technically challenging screw placements with limited margin for error, particularly in pediatric patients with smaller and more variable anatomy [19]. Common complications included persistent symptoms (19.5%), defined as unresolved pain, limited range of motion, or residual neurological deficits that were still present at final follow-up despite achieving radiographic fusion. Other complications included deep wound infections (9.76%) and failed fusion (9.76%). Techniques such as C1-C2 wiring and transarticular screws showed higher complication rates, primarily due to graft resorption and hardware issues.

In contrast, advanced methods like C2 translaminar screws demonstrated superior outcomes with no complications, likely attributed to their enhanced biomechanical stability, reduced risk of hardware-related issues, and improved compatibility with pediatric anatomy [17]. Pediatric C2 translaminar screw fixation also carries a lower risk of vertebral artery injury and serves as a valuable alternative when anatomical constraints preclude the use of C1-C2 transarticular or C2 pars screws. Moreover, the O-C2 translaminar screw construct can reduce anterior pathology when it is present and reducible and has demonstrated a promising first-time construct fusion rate of 92% [20]. Technical challenges, including graft resorption and pin site infections, further underscore the importance of precision in surgical execution and advancements in instrumentation.

The anterior approach (27.4%, n=17) primarily involved odontoid screw fixation, resulting in an 88.2% fusion rate. Complications included failed fusion (5.88%) and one mortality (5.88%) due to neurovascular injury. Strategies to mitigate these risks include thorough preoperative imaging to assess anatomical variations, precise intraoperative navigation to minimize screw misplacement, and postoperative protocols focused on early detection and management of complications. Transient swallowing difficulty, reported in Wang et al. [14], resolved without intervention. Despite these challenges, the anterior approach remains effective for addressing anteriorly displaced fractures, provided meticulous preoperative planning and intraoperative precision are maintained.

The combined approach (6.5%, n=4) yielded the most favorable outcomes, achieving a 100% fusion rate with no complications or mortality. Techniques involved anterior decompression, such as transoral odontectomy, followed by posterior fixation with C1-C2 screws. This approach was efficient in complex cases requiring enhanced stability [13,4]. These findings support the role of combined approaches in addressing severe dislocation or deformities.

Overall, this review corroborates existing literature, indicating consistently high fusion rates but variable complication profiles across techniques. The choice of surgical approach should consider patient-specific factors, including the type of fracture, age, and comorbidities. Recent advancements, such as C1-C2 transarticular and C2 laminar screws, hold promise for reducing complications and improving outcomes. Further investigations, particularly those assessing long-term skeletal development, postoperative deformity risk, and data from broader, multi-institutional pediatric cohorts, are essential to refine surgical strategies and enhance long-term care. In addition, the persistence of challenges such as neurological decline and failed fusion highlights the ongoing need to refine surgical techniques and postoperative care protocols, particularly since varying definitions of complications across studies may lead to inconsistencies in reported outcomes.

Limitations

This review is constrained by a limited sample size of 62 pediatric cases and considerable variability among the included studies. Additionally, most studies were retrospective, which may introduce limitations such as incomplete data and inconsistent methodology. Publication bias also remains a concern, as studies with favorable outcomes are more likely to be published, potentially skewing perceptions of surgical success. Variability in patient populations, surgical techniques, and outcome definitions further limits the generalizability of findings. Moreover, the lack of long-term follow-up in some studies hinders the assessment of fusion durability and the detection of delayed complications, such as adjacent segment disease. Limited comparative data, notably the absence of head-to-head trials comparing anterior, posterior, and combined approaches, restricts definitive conclusions about the superiority of any one technique [2].

Additionally, correction for multiple comparisons was not performed. We acknowledge this limitation in the interpretation of borderline p-values. To address these limitations, future research should focus on prospective, multicenter studies with larger cohorts and standardized protocols. This includes clear definitions for key surgical outcomes, such as fusion success and complications, as well as the adoption of core outcome measures to reduce reporting variability and improve the reliability and applicability of findings.

## Conclusions

This systematic review highlights the importance of tailoring surgical approaches to individual patient needs, taking into account both anatomical and pathological factors. While the posterior approach remains the most utilized and versatile option, the anterior and combined approaches provide valuable alternatives in select cases. Recent innovations such as 3D intraoperative navigation, robotic-assisted surgery, imaging guidance, and minimally invasive techniques hold significant promise for enhancing surgical precision and safety in pediatric odontoid fracture management.
